# The Facilitators and Barriers of the Implementation of a Clinical Decision Support System for Breast Cancer Multidisciplinary Team Meetings—An Interview Study

**DOI:** 10.3390/cancers16020401

**Published:** 2024-01-17

**Authors:** Lejla Kočo, Carmen C. N. Siebers, Margrethe Schlooz, Carla Meeuwis, Hester S. A. Oldenburg, Mathias Prokop, Ritse M. Mann

**Affiliations:** 1 Department of Imaging, Radboud University Medical Center, Geert Grooteplein Zuid 10, 6525 GA Nijmegen, The Netherlands; 2 Department of Surgery, Radboud University Medical Center, Geert Grooteplein Zuid 10, 6525 GA Nijmegen, The Netherlands; 3 Department of Radiology, Rijnstate, Wagnerlaan 55, 6815 AD Arnhem, The Netherlands; cmeeuwis@rijnstate.nl; 4 Department of Surgery, The Netherlands Cancer Institute (Antoni van Leeuwenhoek), Plesmanlaan 121, 1066 CX Amsterdam, The Netherlands

**Keywords:** breast cancer healthcare pathway improvement, clinical decision support systems, electronic medical records, stakeholder involvement, workflow efficiency improvement

## Abstract

**Simple Summary:**

This interview study delves into the potential of using AI-based clinical decision support systems (CDSSs) during meetings focused on multidisciplinary team meetings (MDTMs) for breast cancer. The goal was to pinpoint the obstacles and to aid in implementing such a system within these meetings. Through 24 interviews with breast cancer team members across three hospitals, key insights emerged. Those involved showed interest in integrating CDSSs into their workflow, foreseeing benefits like enhanced data visualization, time-saving functionalities, and improved documentation. However, concerns lingered around data connectivity, the accuracy of suggestions, and the risk of losing the human touch in decision making. Overall, this research reveals the curiosity among clinicians to explore CDSS benefits but acknowledges the complexity of integrating these systems, offering insights to potentially streamline future implementation processes.

**Abstract:**

Background: AI-driven clinical decision support systems (CDSSs) hold promise for multidisciplinary team meetings (MDTMs). This study aimed to uncover the hurdles and aids in implementing CDSSs during breast cancer MDTMs. Methods: Twenty-four core team members from three hospitals engaged in semi-structured interviews, revealing a collective interest in experiencing CDSS workflows in clinical practice. All interviews were audio recorded, transcribed verbatim and analyzed anonymously. A standardized approach, ‘the framework method’, was used to create an analytical framework for data analysis, which was performed by two independent researchers. Results: Positive aspects included improved data visualization, time-saving features, automated trial matching, and enhanced documentation transparency. However, challenges emerged, primarily concerning data connectivity, guideline updates, the accuracy of AI-driven suggestions, and the risk of losing human involvement in decision making. Despite the complexities involved in CDSS development and integration, clinicians demonstrated enthusiasm to explore its potential benefits. Conclusions: Acknowledging the multifaceted nature of this challenge, insights into the barriers and facilitators identified in this study offer a potential roadmap for smoother future implementations. Understanding these factors could pave the way for more effective utilization of CDSSs in breast cancer MDTMs, enhancing patient care through informed decision making.

## 1. Introduction

Breast cancer is a highly complex disease which requires combined modality treatment. To facilitate this type of treatment, multidisciplinary team meetings (MDTMs) are an indispensable component of the standard breast cancer care pathway [1,2]. The goal of breast cancer MDTMs is to ensure proper evidence-based management of breast cancer patients. During MDTMs, specialists from different disciplines involved in breast cancer treatment review patient cases to determine the diagnosis and propose management plans [3].

However, MDTMs are considered time consuming and expensive [4,5]. Moreover, the increasing number of breast cancer patients worldwide can potentially disrupt the current structure and organization of breast cancer MDTMs [6]. In other words, the combination of high disease complexity, high patient loads and high costs requires hospitals to practice efficient and well-organized breast cancer MDTMs. Therefore, optimizing the structure and efficiency of MDTMs is important for successful breast cancer care [7].

The improvement of healthcare delivery is one of the main goals of clinical decision support systems (CDSSs). CDSSs are currently under development for many different diseases and use a combination of clinical knowledge, patient information and other health-related information such as guidelines to support healthcare professionals during various stages of the clinical decision-making process [8]. By providing evidence-based or best practice suggestions, CDSSs may increase the quality and efficiency of MDTMs, consequently increasing the quality of care [9]. Several different types of CDSSs are available for breast cancer, focusing on a variety of aspects, from risk or recurrence to determining available therapeutic options [10]. Some CDSSs even incorporate artificial intelligence (AI) in order to manage and analyze the data in a more advanced manner, adding another dimension of facilitators and barriers, such as user trust and legal or ethical considerations, to the implementation process [11,12].

However, a recent systematic review has shown the overall use of CDSSs was limited to 34.2%. Furthermore, evaluating the available patient data quality needed as input and identifying behavior change barriers were found to be crucial factors associated with actual use of CDSSs [13]. General barriers of implementation range from technical challenges to usability issues [14,15]. Therefore, expectations and concerns of healthcare professionals are essential aspects to consider during the development and implementation phases of CDSSs [16]. The viewpoints of healthcare professionals on CDSSs and their potential implementation, especially in the context of breast cancer care, remain unexplored.

Therefore, the scope of this study is to identify the areas where CDSSs can be of added value. Intended end users of breast cancer CDSSs (breast cancer clinicians) were interviewed in this study. Their perspectives may aid in the development of CDSSs and improve the usefulness of such programs in clinical practice. This study aims to identify the barriers and facilitators of the idea of implementing a CDSS during breast cancer MDTMs.

## 2. Materials and Methods

### 2.1. Design

This study is a follow-up study of our study regarding the improvement of breast cancer MDTMs [17]. A qualitative, semi-structured interview approach was chosen as it provides an effective way of exploring the views and opinions of breast cancer specialists regarding the implementation of a CDSS during breast cancer MDTMs.

### 2.2. Participants and Procedures

As preparation for this study, breast cancer MDTMs were observed to obtain an understanding of the current approach and organization.

The semi-structured interviews were carried out by the first author (L.K.) either face-to-face, by phone or by video call. To ensure a representative group of participants, the core team members of breast cancer MDTMs from a regional hospital, an academic hospital and a specialized cancer institute were included, i.e., surgeons, oncologists, radiologists, radiation oncologists, pathologists, and nurse specialists. One core member of the breast cancer MDTM in each hospital was approached with the request to participate in this study. From here, other core members of the MDTMs were included until at least one healthcare professional per core discipline was interviewed. By including various disciplines, participants with different tasks and responsibilities during the MDTM were included to ensure a range of perspectives.

The three participating hospitals in this study used either Chipsoft (HiX) or EPIC electronic medical record (EMR) systems and used separate programs for reporting radiology and pathology results, which were in turn uploaded to the respective EMR systems. The first overview page in each patient’s file showed a selection of summarized recent patient information. Both EMR systems were structured in sections that were designated to store reports and images for several disciplines such as general patient information, pathology, radiology, and surgery. Furthermore, logistical information was presented in separate tabs (e.g., planned appointments, requested procedures, and research or trial information).

To ensure consistency between interviews, an interview guide was developed (Supplementary File S1). The following topics regarding CDSSs were covered during the interviews: current perceived issues during breast cancer MDTMs, the CDSS functionalities, expectations, doubts, and potential barriers of implementation. In all interviews, the conversation was started with a description of the goal of the interviews and an example CDSS, in order for the participants to give their personal opinions on the described general and more advanced CDSS possibilities. All interviews lasted between 25–45 min. Participants gave permission to audio-record and anonymously transcribe the data verbatim. The transcript of each interview was summarized and presented to the corresponding participant for their confirmation of the content. Usually, interviews are performed until data saturation is reached. In this case, data saturation was reached shortly before the final interviews, but despite this, all intended participants were interviewed.

### 2.3. CDSS Features Example

A CDSS was used as an example to illustrate the possibilities of such a system and support discussion [18]. The CDSS example consisted of mockup CDSS pages with specific functionalities modeled with (fictional/scrambled) patient cases. The interviewer described the program as follows: the CDSS is a separate program with multiple tabs, each focusing on a separate aspect ranging from patient characteristics to suggestions that are automatically generated based on the available data. A connection between the AI-based CDSS and the electronic medical records (EMRs) is established which allows the CDSS to use relevant patient characteristics, results, and other data. The CDSS would be able to generate a clear overview of the available data in a concise summary and a data completeness check. Furthermore, it is also possible to view radiological images. Using AI in the CDSS would allow the system to support healthcare professionals by generating a template for MDTM reports, diagnostic or treatment suggestions based on guidelines and the automatic matching of each eligible patient to an open clinical trial. A healthcare professional could use different tabs of the CDSS during different stages of the MDTMs, such as preparation and the MDTM meeting itself. For example, the patient summary could be used during the preparation, while the guideline suggestions may support healthcare professionals in formulating evidence-based advice.

### 2.4. Data Analysis

A standardized approach for interview analysis, the framework method, was used to develop an analytical framework for analysis to avoid researcher bias [19]. First, the researchers familiarized themselves with the data by transcribing and/or reviewing the interviews to gain an extensive understanding of the content. Second, key concepts, topics and patterns were identified from the data resulting in the thematic framework. Third, data segments from the transcripts were systematically tagged and coded according to the established thematic framework. This step was performed by two researchers (L.K. and C.S.) independently and compared. Disagreements were to be resolved through discussion. The fourth step consisted of organizing and summarizing the coded data. Finally, the patterns and connections within and between themes were explored and summarized. 

The results were added in a table using the structure of the measurement instrument for determinants of innovation (MIDI) created by Fleuren et al. [20]. The MIDI determinants were solely used to structure the results in a clear but relevant overview. All statements from the interviews were coupled to the corresponding determinant from the MIDI. As described by the MIDI guide, only determinants relevant to this study were included in the table. The included determinants are as follows:

Innovation: correctness (2), completeness (3), complexity (4), compatibility (5), observability (6), relevance for client (7); User: personal benefits/disadvantage (8), outcome expectation (9), professional obligation (10); Organization: material recourses and facilities (24); and Socio-political context: legislation and regulation (29).

In the results section, all relevant topics are coupled to the corresponding determinant with the number of times the topic was mentioned during the interviews. The programs “Express Scribe” (transcription) and “Atlas.ti” (coding) were used to analyze the audio recordings and transcripts obtained from the interviews.

## 3. Results

### 3.1. Participant and Hospital Overview

In total, twenty-four breast cancer specialists participated in this qualitative study, of which nineteen were female (79%) and five were male (21%) (Table 1). From the general hospital, six specialists were approached, and in both the academic hospital and the cancer institute, nine specialists each were included. All participants attended the breast cancer MDTMs at least weekly.

### 3.2. Interview Results

**Figure 1:**
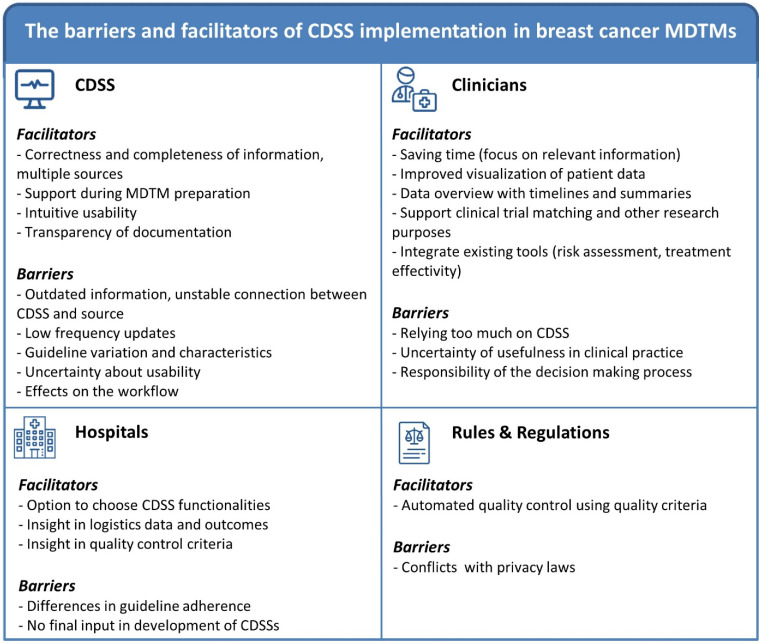
Overview of all identified barriers and facilitators of breast cancer MDTMs.

All statements, as mentioned, given by the participants are displayed in Table 2, Table 3 and Table 4. All results are subdivided according to the following MIDI categories: innovation (CDSS), user (breast cancer clinicians), organization (hospitals) and socio-political context (laws and regulations). Figure 1 summarizes all barriers and facilitators as identified by the participants of this study.

#### 3.2.1. Results Associated with Innovation (CDSS) Use and Implementation

The following topics of interest concerning the CDSS emerged from the interviews: (1) patient information visualization, (2) clinical trial matching and (3) guideline-based suggestions. These results concerning CDSS content and functionalities are also shown in Table 2.

**Table 2:** Overview of interview results using determinants from the MIDI to classify facilitators and barriers as specified by the participants—Category: Innovation.

Innovation: CDSSs		
MIDI Determinant	Facilitators	Barriers
**Correctness (2)**	- Guaranteed correctness of displayed information by the CDSS (*n* = 4) - A CDSS that reduces error rate (*n* = 4)	(1) Clinical trial matching would be useful - for own hospital (*n* = 9) - for other hospitals (*n* = 1) - when trials are included on international level (*n* = 1) - for smaller trials with very specific inclusion criteria or small patient populations (*n* = 1) - when many trials are ongoing simultaneously (*n* = 1) - to speed up patient matching to trials (*n* = 1) - since trials participation varies on national level (*n* = 1) - since it currently depends on attendees of MDTMs (*n* = 4) (2) Showing specific diagnostic and treatment suggestions tailored per patient are useful: - when personalized and to check the advice as formulated by clinicians (*n* = 6) - for documentation of decision and visualizing guideline adherence (*n* = 5)
**Completeness (3)**	- Integrating information from multiple sources into one clear overview (*n* = 14) - A CDSS should display complete patient information clearly and fast (*n* = 9) - Automated completeness checks of patient information (*n* = 2) - Ability to show pathology and radiology results (*n* = 2)	- A CDSS that is unable to provide decision support for outliers and exceptional cases (*n* = 1)
**Complexity (4)**	- With a clear information overview, verification of patient information would be facilitated (*n* = 1)	- There are different guidelines available, some disciplines even regional, so challenging to implement (*n* = 2)
**Compatibility (5)**	- MDTM preparation is time consuming but essential to ensure MDTM efficiency (*n* = 10) - Supporting clinicians in existing tasks such as preparation of MDTMs (*n* = 4) - CDSS that is easy to use (*n* = 3)	- There are already too many systems in use as it is (*n* = 3) - Unwanted functionalities or pop-ups, slowing down the workflow (*n* = 3)
**Observability (6)**	- Improving transparency of documentation, additional information (*n* = 4)	- No guarantee of how CDSS will affect workflow and whether the functionalities will be beneficial (*n* = 2)
**Relevance for client (7)**	(1) Clinical trial matching would be useful - for own hospital (*n* = 9) - for other hospitals (*n* = 1) - when trials are included on international level (*n* = 1) - for smaller trials with very specific inclusion criteria or small patient populations (*n* = 1) - when many trials are ongoing simultaneously (*n* = 1) - to speed up patient matching to trials (*n* = 1) - since trials participation varies on national level (*n* = 1) - since it currently depends on attendees of MDTMs (*n* = 4) (2) Showing specific diagnostic and treatment suggestions tailored per patient are useful - when personalized and to check the advice as formulated by clinicians (*n* = 6) - for documentation of decision and visualizing guideline adherence (*n* = 5)	- The usefulness of guideline suggestions depends on the specificity of guidelines per discipline (*n* = 1)

##### Visualizing Patient Information

According to the participants, the preparation of MDTMs is time consuming but an essential factor for the efficiency of MDTMs (*n* = 10). Currently, case summaries with information necessary for MDTM discussion are not available for healthcare professionals prior to the MDTM. Therefore, each participant prepares the cases on the MDTM list individually by looking up all relevant information to answer the posed question. Since patient information is stored in different places in the EMR and across several different programs, clinicians need to navigate through different environments to find the specific patient information they need. The necessary information is similar for most cases, but it is not easily found. Therefore, participants considered a CDSS as a possibility to support their preparation tasks (*n* = 4), for example, by automatically generating a clear patient summary. Also, a CDSS could facilitate efficient discussions during the MDTM by providing a clear visual overview of all relevant information in timelines and schematic overviews (*n* = 14), integrating information from multiple sources into one program (*n* = 14) and decreasing distractions caused by irrelevant information in the patient file (*n* = 2). Moreover, integrating shortcuts to view pathology and radiology resulting in the CDSS would eliminate the need to switch between different programs (*n* = 2). Using a CDSS that can partially automate the documentation of MDTM decisions is expected to improve efficiency and potentially reduce errors in the reporting process (*n* = 8). Consequently, by improving and simplifying the documentation process, considerations during discussions could be made more transparent (*n* = 2). Important requisites for using the CDSS during MDTMs, however, would be the display of reliable and correct information (*n* = 4) and a fluent workflow that matches current clinical practice to avoid becoming ‘yet another program to use’ (*n* = 3).

*“Maybe you can indeed have that general overview in one screen and then show a summary or conclusion. And then have the advice summarized on another screen. So, you’re not scrolling through it. What you naturally hope for is to make it even more efficient than it is now. So that all the data is right there at one glance, without the need for searching or extra effort. That would be great.”*

(Breast cancer specialized nurse)

Highlighting incomplete information of scheduled patients was also raised as a potential point for improvement. This is due to either having incomplete results of performed diagnostic tests or available but not yet visible results in the EMR. Incomplete patient information during the MDTM or discussing preliminary MDTM advice is considered inefficient, since the patient will most likely be rediscussed in the next MDTM (*n* = 10). The CDSS could support the workflow by automatically determining the completeness of patient information (*n* = 2) and simplifying the search and verification of patient information (*n* = 2). The main concern of participants was the data connection between the CDSS and the currently used hospital systems. Furthermore, the EMR is not always synced in real-time with other programs leading to a delay in the depiction of the latest results in the EMR. Some departments use their own system, which is coupled with the EMR; therefore, this is something that should be addressed. In the end, a CDSS should be easier and more intuitive to use than the current EMR systems (*n* = 3).

*“It’s quite inconvenient not to have certain information you need readily available in the EMR, leading to a lot of searching. Everyone starts searching. That can be confusing at times. Even though that information is actually already available in another system but not shown in the EMR. Ideally you want it to be as up-to-date and accurate as possible.”*

(Pathologist)

##### Identifying Potential Clinical Trial Participants

Clinical trials are one of the focus points of case discussions during MDTMs. The number of ongoing research projects and trial participation rates differ per hospital. Identifying patient eligibility for a study depends on the extent to which MDTM members are aware of all inclusion criteria or on the presence of a researcher during the MDTM. Because some studies require very specific and rare patient subgroups, eligible patients can easily be overlooked. Therefore, participants stated that clinical trial matching is a factor that could be improved by using a CDSS (*n* = 9). Automatic identification of potential trial candidates could support the MDTM. With the aid of a CDSS, all eligible patients for clinical trials and studies could be considered for participation equally.

*“Currently it is our responsibility to decide whether a patient is eligible for a study. If a system could pick that up and say, ‘Hey, I see that this patient qualifies for this particular study.’ I think that would be extremely helpful because it’s a shame to forget these types of things.”*

(Radiotherapist)

##### Automatic Suggestions for Diagnostic Procedures and Treatment

In all three participating hospitals, the MDTM advice is based on the Dutch national breast cancer guidelines, with slightly varying local guidelines per hospital for certain specific treatments such as systemic therapy or radiotherapy. For deviation from the guidelines, considerations made during the discussions are documented in the MDTM report. A potential functionality of CDSSs to support healthcare professionals in treatment decision making during the MDTM is to compare patients’ medical status to the current guidelines and propose personalized suggestions accordingly.

Participants’ attitude towards this functionality ranged between positive and tentative. On one side, healthcare professionals are familiar with the guidelines from their own discipline, therefore often not explicitly mentioning it during the MDTM discussions. Using a CDSS which always shows the steps for each patient according to the guidelines could make the decision-making process more transparent, since the given advice and the advice according to guidelines would always be documented together (*n* = 5).

*“I do think it’s good to take a step back to what the standard (guideline) is, when we opt for something different. Because, when you look back at your notes, they’re very concise. If you weren’t present, you could still understand why we decided on certain recommendations. I know that when I haven’t been present myself, sometimes the notes are so concise that I wonder: which steps did they take. Yes, I believe it does make it clearer when you present it that way. And it’s also more comprehensible for those who weren’t there.”*

(Breast cancer specialized nurse)

On the other side, a participant stated that not all patients can or should be treated strictly according to the guidelines (*n* = 1). In certain cases, it can potentially be better to deviate from the protocol, and in some patient subgroups, guidelines are not even available or applicable. A CDSS was suggested to discourage this important option. Additionally, concerns were raised on how often and fast a CDSS would be able to implement guideline updates (*n* = 5). As guidelines are already running behind clinical practice when they are published, a delay in updating them in the CDSS increases that gap even more.

Importantly, whether clinicians find the suggestions helpful mainly depends on the way the suggestions are presented by a CDSS and how detailed they are (*n* = 6). It should serve a role as a final check for the given advice and would especially be considered useful if the suggestions are completely personalized to each patient case (*n* = 13).

*“Looking at the breast cancer guidelines for oncologists, it mainly states, ‘consider this’ and ‘discuss with the patient.’ And those are actually the two most important things. So, yes, it remains a bit vague. It’s more about leaving various different options that could be considered. You might be able to include guidelines for systemic therapy in a CDSS. But I think the nuance of discussing or considering will likely remain, and it might not significantly change the policy, although it’s more convenient to have it already there on screen, so you don’t have to type it.”*

(Oncologist)

#### 3.2.2. Results Associated with Users (Breast Cancer Clinicians)

Table 3 summarizes potential effects of the CDSS on breast cancer clinicians.

**Table 3:** Overview of interview results using determinants from the MIDI to classify facilitators and barriers as specified by the participants—Category: User.

User: Breast Cancer Clinicians		
MIDI Determinant	Facilitators	Barriers
**Personal benefits and disadvantage (8)**	Time-saving functionalities of CDSS according to clinicians: - Improved visualization of patient information such as summary and timeline (*n* = 14) - Not having to extensively search for information and copy/paste data (*n* = 5) - Clinical trial matching (*n* = 3) - There is less additional (non-relevant) information to look through in a CDSS that provides an automatic summary (*n* = 2)	- A CDSS that takes over too many tasks may lead to clinicians relying too much on a program (*n* = 5)
**Outcome expectation (9)**	- Using the CDSS for research purposes (*n* = 1) - Using CDSS for automatic calculation of estimated treatment effectivity (n = 1)	- Clinicians have no specific expectation of CDSS and have to wait and see what the outcomes will be (*n* = 3) - Some aspects of MDTMs simply cannot be improved by the implementation of CDSSs (*n* = 1) - Guideline suggestions by a CDSS will not influence decision-making results (*n* = 4)
**Complexity (4)**		- Risk of CDSS taking over standard workflow and preparation, responsibility over whole decision-making process (*n* = 4)

One of the main benefits of using a CDSS for clinicians would be to save time compared to the current workflow. Participants shared different ways in which this goal could potentially be accomplished. Improved visualization of patient information with summaries and timelines were mentioned most often (*n* = 14), since it would reduce the amount of time clinicians have to search and copy–paste information (*n* = 5). Also, there would be less non-essential information they have to look through when searching specific pieces of information (*n* = 2). Finally, some participants stated that the functionality for clinical trial matching could save time (*n* = 3).

*“Maybe someday you would want an MDTM report to be automatically generated. Where you can specify input that really matters, and then you already have a sort of pre-generated report to work in. Because then you only need to type the advice. And what you notice in this EMR system is that some people do a complete copy-paste from the initial consultation and keep repeating it continuously in other reports. I want to know what it’s about, without very vague language. I just want to know relevant information for that report, briefly, the characteristics.”*

(Surgeon)

*“Sometimes people end up repeating things during the MDTM. So sometimes, all these ideas are put on the table, and then they say we should do this now, we should do that now. And then it’s discussed for a bit, and people start suggesting doing this or that again. And then no decision is made. So, I think having some form of clinical decision-making tool would be quite helpful.”*

(Radiologist)

However, a CDSS should not take over too many tasks since that may lead to clinicians automatically relying too much on the system instead of on their own knowledge (*n* = 5). In line with this, participants felt they had an obligation and responsibility for the whole decision-making process and therefore saw using CDSSs as a risk (*n* = 4).

*“Automatic suggestions could also be helpful to check, but you might have to do it in a certain order. First making your own recommendation, and then seeing what the CDSS suggests based on guidelines, and then discussing that. But if you let only the computer, make a suggestion first and then the clinician, the clinician might get lazy because after ten times, they’ll just agree with the computer.”*

(Pathologist)

Participants found it difficult to identify specific expectations of the outcomes of using a CDSS (*n* = 3), since it was also felt that some aspects of the workflow and MDTMs could not be improved by simply using a CDSS (*n* = 1). Some participants stated that they do not expect that functionalities such as suggestions based on guidelines will influence the decision-making process significantly (*n* = 4). However, two suggestions for other potential functionalities and outcomes from using a CDSS were made. For example, one suggestion was made to integrate online risk assessment or prediction tools to optimally manage treatment options also according to predicted effectiveness (*n* = 1). Another participant suggested to also use the knowledge in the CDSS for research purposes to understand more about treatment trends and effectiveness (*n* = 1).

*“I find it hard to have an expectation about the CDSS now. What you naturally hope for is to make it even more efficient than it is now. And how that will actually look, well, I can’t really say for sure right now.”*

(Breast cancer specialized nurse)

*“If there’s a pattern in the data that we’re not picking up ourselves, then I think it would be fantastic if we could use deep learning techniques to extract new information to advance our knowledge. I don’t necessarily see this as support for an MDTM, but more in the general development of our healthcare and knowledge. We make estimations based on statistics as best as we can to determine who needs adjuvant treatment or not, but it’s still very rough. If there’s an artificial intelligence or deep learning system that can do it better, I’m all for it. But again, that’s more of a research setting than an CDSS or MDTM setting.”*

(Oncologist)

#### 3.2.3. Results Associated with Organization (Hospitals) and Socio-Political Context

Some identified facilitators and barriers were related to organizations and socio-political context (Table 4, (4.1) and (4.2)).

**Table 4:** Overview of interview results using determinants from the MIDI to classify facilitators and barriers as specified by the participants—Category: organization (4.1) and socio-political context (4.2).

**4.1 Organization: Hospitals**		
**MIDI Determinant**	**Facilitators**	**Barriers**
**Material resources and facilities (24)**		- Guideline adherence differs per hospital; some hospitals treat less conservatively (*n* = 4)
**Performance feedback (28)**	- Hospitals/clinicians can choose which functionalities to use in CDSS (*n* = 2) - Having insight in logistical data and quality criteria outcomes (*n* = 2)	- If specialists (end users of CDSS) do not have a final say during the development of a CDSS (*n* = 1)
**4.2 Socio-Political Context: Quality of Care Indicators**		
**MIDI Determinant**	**Facilitators**	**Barriers**
**Legislation and regulations (29)**		- CDSS is conflicting with privacy laws (*n* = 1)

Factors such as workflows, protocols and patient population differ between hospitals. For this reason, the possibility to choose which functionalities of a CDSS each hospital is going to use individually would be important (*n* = 2). The inclusion of end-users during the development phase of a CDSS is appreciated (*n* = 1). Furthermore, quality criteria of care apply to all Dutch hospitals and are continuously being monitored and evaluated. Participants mentioned that having insights into the logistical processes (such as waiting times for certain treatments) and current status of the quality criteria in their hospital would be useful (*n* = 2). When considering the suggestions regarding the guidelines functionality, participants stated that guideline adherence can differ between hospitals. For example, high rates of clinical trial participation or treatment in a specialized breast cancer hospital could result in treating patients less conservatively and would thus mean deviating from guidelines more often (*n* = 4). Finally, an important concern to address would be the conflict of CDSSs (using patient data) with Dutch privacy laws (*n* = 1).

*“Having unwanted pop-ups from the national guideline saying, ‘do this or that’, no. Being able to select the functionality that you want to use is better. As soon as there are functions that only give you trouble, making you have to close them, then it works against you because it becomes a nuisance. That’s a shame considering the other functions that might actually be beneficial.”*

(Radiotherapist)

## 4. Discussion

### 4.1. Summary of Evidence

This study provides an overview of the barriers and facilitators of the potential implementation of CDSSs during MDTMs, as identified by breast cancer specialists. In general, participants were interested in trying CDSSs during their work in order to experience the effect of a CDSS on workflow in clinical practice. According to the participants, breast cancer MDTMs are already considered to be quite streamlined with the present organizational structure. Combined with a theoretic description of a CDSS, participants found it challenging to identify concrete expectations on the potential benefits of using a CDSS in clinical practice for breast cancer care. The concerns that were voiced were mainly related to the quality of the data connections between a CDSS and other programs, the updating of source materials such as guideline protocols, the accuracy of the provided automatic suggestions, and losing the human component of the decision-making process.

### 4.2. General Status of CDSS Implementation in Clinical Practice

The first CDSSs date back to the 1970s in the form of alerts and reminders for clinicians. However, to this day, widespread implementation in clinical practice has not yet been achieved, since challenges and barriers still seem to persist [8,13,21]. This could be related to the fact that there is very little evidence available for the positive effects of using a CDSS in clinical practice [8,10,21,22,23,24]. Furthermore, the definition of the term CDSS is very broad and can include a wide range of features and functionalities with different applications, proving a challenge for proper evaluations and comparisons of studies [10,23]. For example, breast cancer CDSSs can be subdivided in (1) risk calculators for recurrence/death or survival per specific treatment type or (2) by providers of suggestions of available therapy options based on specific patient characteristics [10]. A focus group study showed similar barriers and facilitators to the results obtained in this study, e.g., uncertainty of responsibility over decisions, reduced clinician autonomy and potential extra workload due to extra reminders or notifications, the importance of self-control over the frequency and content of these reminders, and improved work processes [25]. While regarding the CDSS, related aspects such as existing hospital systems, technical challenges and laws must be considered. Additionally, the changes that could come with the implementation of CDSSs to general aspects of MDTMs, such as the decision-making process, workflow, and collaboration between attendees, should also be considered in the context of users.

### 4.3. Barriers and Facilitators: CDSS and Processes

Factors such as data quality, update strategies, the accuracy and nature of the suggestions, and the source of the CDSS suggestions were identified as both barriers and facilitators in this study. When accurate, these factors would be appreciated, but concerns were raised regarding the ability of CDSSs to deliver high-quality data promptly and update regularly. The potential benefits of implementing a CDSS, like effectively integrating patient information from various sources accurately and comprehensively, could enhance a positive attitude among future users towards the implementation of a CDSS. Likewise, the idea that a CDSS could potentially provide a more streamlined workflow acts as a facilitator [26].

Several other studies have identified similar potential barriers related to system and process aspects [8,10,21,27]. Other potentially hindering factors are limited computer literacy, lack of system and content maintenance, lack of interoperability with existing software already in use (such as EMRs, pathology systems or clinical imaging software), and financial challenges [8,10,24]. For example, for suggestions of CDSS that could be followed in clinical practice, all other treatment options should also be available while showing the rationale behind this choice. This requires the loading of complete local guidelines into the CDSS [27,28]. Another drawback of AI-based CDSSs is that the exact sequence of reasoning and information used to come to the final suggestions remains unknown to the user, also known as the ‘black box effect’. Since the reasoning behind CDSS suggestions is not transparent, trust in these systems could be more challenging to obtain [27].

### 4.4. Barriers and Facilitators: Clinicians

In order to achieve CDSS implementation in clinical practice, it is essential to obtain acceptance and trust in the CDSSs functioning and suggestions from the targeted end-users [10]. From a clinicians’ point of view, the usability and efficiency of a CDSS requires a balance between workflow improvement (by means of better retrieval and presentation of information), while not exposing them to increased workload or alert fatigue [8,22]. Currently, clinicians face a significant challenge due to the fragmentation of patient information across various programs, leading to time-consuming searches for specific pieces of information, spending valuable time and effort which could be otherwise dedicated to more relevant tasks. The results show that the facilitators that were identified in this study are mainly related to improving these issues. 

However, the results also showed that apprehension about using a CDSS is still present among the participants. Part of their worries were related to what it would mean for their workflow and their patients if a CDSS would be implemented in clinical practice, specifically regarding suggestions made for certain (complex or rare) cases where guidelines are vague or non-existent. Furthermore, worries existed that CDSSs would take over too many tasks, changing the roles and responsibilities within the decision-making process. Furthermore, a potential solution for not having to use numerous different programs could be to integrate the CDSS functionalities into EMRs. One study has shown a potential moderate improvement of morbidity outcomes but no improvement in survival when assessing the effectiveness of a CDSS integrated with EMRs [23]. Finally, the involvement of end-users during the developmental and implementation phase of CDSSs is welcomed by participants in order to specifically develop functionality according to actual expectations, needs and compatibility with current workflows in clinical practice [29,30,31].

### 4.5. Rules and Regulations

The implementation of CDSSs in healthcare systems faces multiple challenges regarding laws and regulations, as the results have suggested concerns from healthcare professionals regarding privacy and data sharing between systems. First of all, there was a concern regarding how to ensure compliance with (inter)national data/privacy protection laws, given the sensitive nature of patient information handled by CDSSs. Additionally, legal liability and accountability in regulatory frameworks should be established, in case of errors or adverse effects, before widespread use [8,32]. Moreover, the adherence of CDSSs to validation and quality assurance standards requires attention. Currently, the verification of CDSS suggestions is challenging, which complicates the evaluation process. Evaluations of AI-based CDSSs mainly focus on the performance of algorithms [22,27]. Thus, this highlights the need for dedicated verification and validation tools for practical implementation [27,33]. Lastly, current interoperability standards vary across different programs, leading to complexities in data exchanges between different systems—a fundamental challenge that must be addressed to enable proper implementation of CDSSs. However, this issue is not specific to CDSSs but to any medical program that requires connections to others [8,34,35].

### 4.6. Patient Perspectives

The healthcare professional perspective is not the only one that should be considered. Patient perspectives are important as well when considering the widespread implementation of CDSSs. Ultimately, the aim of CDSSs is to enhance cancer care by supporting healthcare professionals in decision making and workflow efficiency, also benefiting patients. Integrating patient preferences within the systems could further facilitate shared decision making and enhance the way patients are included in the decision-making process. However, certain concerns should be considered, such as the legal concerns mentioned in the previous paragraph. Worries about CDSSs replacing healthcare professionals in the decision-making process might arise. Advice for patients is not solely based on the information stored in the EMR but also on informal information from the patient collected during consultations [22]. A study showed that rather than replacing clinicians, a CDSS provides recommendations that are consistent with typical clinical decisions. They emphasized the diversity of factors influencing the decision-making process and therefore also the importance of optional CDSS suggestions [28]. Yet, studies indicate the potential for improved outcomes when clinicians utilize CDSS for decision making [36,37].

### 4.7. Strengths and Limitations of This Study

The findings of this study should be considered in light of some limitations. Oncology CDSSs have not been widely implemented yet in clinical practice. Additionally, the term CDSS is very broad and can be used for a range of different programs developed for decision support purposes on several levels. The participants based their opinion and comments solely on a verbal description of an example CDSS, since a full demonstration or test program was not available. It is likely that participants had different ideas about layout and usability of CDSSs and based their opinions on different perspectives. Therefore, the identified facilitators and barriers in this study should be regarded as generally applicable and can potentially be different from those of an actual evaluation of a specific CDSS in clinical practice. Secondly, not all stakeholders in CDSS development were included in this study. Other stakeholders such as hospital managers, patients and IT personnel will encounter CDSSs in clinical practice, and their opinions could also influence the outcome of implementation strategies. Furthermore, the results of this study should be considered within the scope of the Dutch healthcare system, which means they might not be comparable or applicable to the situation in other countries.

In the Netherlands, general guidelines for the organization of oncology MDTMs are in existence [38], according to which each hospital can organize their own MDTMs. Thus, variation in the organizational structure of breast cancer MDTMs exists among different hospitals [17]. The usability of CDSSs across differently organized MDTMs is necessary and should therefore also be considered when studying this topic. A major strength of this study is that specialists from three different types of hospital with different MDTM organizational structures and patient populations were included in order to represent this variation. A further strength is the large diversity of participating specialists. The chances of successful implementation of new innovations are better when end-users are included in early stages of the development [29,30,31]. However, generational differences and years of experience can potentially influence clinicians opinions about digital or AI-based CDSSs [8]. In this study both experienced specialists and residents were included to represent both perspectives. Also, the semi-structured interview method provided the opportunity to explore different opinions and perspectives in more detail when deemed interesting and necessary. Finally, by having two researchers analyze the transcripts independently, most researcher bias was eliminated.

### 4.8. Future Research

In order to achieve the implementation of CDSSs for oncology in clinical practice, additional research is necessary on several levels, including research on functionality, the effects on end-users, the effects on patients and the effects on outcomes. Since the usefulness and additional value of CDSSs appears to vary per cancer type, it is important that these studies are not generalized but tailored to specific health problems including breast cancer care. 

To evaluate the functioning of the system, the user experiences, and the effect on outcomes, RCTs should be performed with CDSSs, as these have not been widely performed yet. Comparative studies between different CDSSs could help us understand which features would be most beneficial for specific cancer types. Longitudinal studies could be considered to understand the long-term impact of CDSS implementation on workflows, patient outcomes and overall care delivery. Furthermore, research on the legal aspects of CDSS implementation should also be performed, as well as studies on the patient and other relevant stakeholders’ perspectives on CDSS implementation.

## 5. Conclusions

CDSSs for oncology have been under development for quite some time. However, despite their potential benefits for workflow and care delivery, their implementation in clinical practice remains scarce. In order to support implementation, barriers and facilitators as identified in this study would best be considered from the start of the development of CDSSs. The implementation of CDSSs is highly dependent on the acceptance and trust of clinicians and patients. In addition, certain challenges, such as access to information sources and data connections between programs, should be overcome to make CDSSs viable for clinical use. Clinicians are, in general, curious and open to seeing the potential benefits of using CDSSs in clinical practice. However, CDSS development and implementation is a multi-faceted and complicated challenge with many different influencing factors and involved stakeholders which should all be included during development and implementation.

## Figures and Tables

**Table 1:** Participant overview in chronological order of interviews [17].

	General Hospital	Academic Hospital	Cancer Institute
**Participant specialism**	1 Surgery resident 1 Surgeon 1 Pathologist 1 Radiologist 1 Radiotherapist 1 Nurse specialist (oncology)	2 Surgeons 1 Nurse specialist (surgery) 1 Oncologist 1 Nurse specialist (oncology) 1 Pathologist 1 Nurse specialist (plastic surgery) 1 Radiotherapist 1 Radiologist	1 Surgeon 1 Surgery resident 1 Surgery fellow 1 Oncologist 1 Pathologist 1 Radiologist 1 Radiotherapist 1 Nurse specialist (oncology)

## Data Availability

The datasets generated and/or analyzed during the current study are not publicly available due to privacy reasons, since the participants would be identifiable, but data relevant to this study are available from the corresponding author upon reasonable request.

## Supplementary Materials

The following supporting information can be downloaded at: https://www.mdpi.com/article/10.3390/cancers16020401/s1, Supplementary File S1: Interview guide.
