# Supplementary material for: The Facilitators and Barriers of the Implementation of a Clinical Decision Support System for Breast Cancer Multidisciplinary Team Meetings—An Interview Study
Source: Cancers (Basel). 2024 Jan 17;16(2):401. doi: 10.3390/cancers16020401 (PMC10813995; doi:10.3390/cancers16020401)
Supplement: Supplementary file 1 [file cancers-16-00401-s001.zip › cancers-2788206-supplementary.pdf]

Supplementary File S1

Interview guide

Introduction to CDSS concept

This study focuses on the barriers and facilitators of the implementation of an artificial intelligence (AI) based clinical decision support system (CDSS) for breast cancer, to be used to prepare and perform multidisciplinary team meetings (MDTMS). The goal of such CDSSs is to give a clear and intuitive overview of all relevant patient information per case. These programs can also have several useful functionalities which could potentially support the workflow during MDTMs. Examples of functionalities are 1) automatic patient summaries, 2) automatically generated treatment suggestions based on guidelines, 3) trial or research participation, 4) automatic documentation and so on. These functionalities could potentially aid in supporting a more efficient workflow surrounding the breast cancer MDTMs. In order to properly implement such CDSS, it is important to identify the barriers and facilitators of implementation according to a healthcare professionals' perspective.

Preparation MDTM

First, I would like to know more about the preparation of the MDTMs. Could you briefly describe how you prepare yourself for the MDTM?

What are the things during preparation of MDTMs that are difficult or time consuming?

How do you think the preparation process could be improved to be more efficient?

MDTM process

How do you assess the current time management of discussions during the breast cancer MDTMs?

How are the Dutch guidelines currently being incorporated during the case discussions (mentioned explicitly or implicitly)?

Is there anything that you would want to change about this method? If yes, how should it be different in the future?

What do you think about the currently used programs during MDTMs (such as the EMR or PACS)?

What are essential components on screen when sharing patient information?

Which functionalities or options do you miss when sharing patient information on the screens?

Which factors do you think determine a successful MDTM? Why do you think that?
What determines the efficiency of a breast cancer MDTM?

Improvement points of MDTMs with support of CDSS

What bottlenecks can you identify regarding the breast cancer MDTMs in both preparation and the meeting itself?

Which factors do you think currently cause delays during breast cancer MDTMs?

Where can time be gained potentially if things were to be improved?

How do you personally think that a CDSS could improve, impact or support the workflow of the whole breast cancer MDTM process?
